# Double-endoscope assisted endoscopic submucosal dissection for treating tumors in rectum and distal colon by expert endoscopists: a feasibility study

**DOI:** 10.1007/s10151-020-02308-4

**Published:** 2020-08-19

**Authors:** A. Ebigbo, G. Tziatzios, S. K. Gölder, A. Probst, H. Messmann

**Affiliations:** Department of Gastroenterology, University Hospital Augsburg, Stenglinstraße 2, 86156 Augsburg, Germany

**Keywords:** Endoscopic, Submucosal, Dissection, Double, Tumor

## Abstract

**Background:**

Colorectal endoscopic submucosal dissection (ESD) is an effective but challenging procedure. To facilitate ESD, several methods that apply traction are available; however, the optimal one remains to be established. The aim of this study was to evaluate the feasibility and safety of the double-endoscope assisted ESD (DEA-ESD) by improving traction to treat complex colorectal lesions.

**Methods:**

Naïve or previously treated lesions in the rectum and sigmoid colon were included. A grasping forceps advanced through a small-caliber endoscope (GIF-XP190N, Olympus Medical Systems, Tokyo, Japan, 5.4 mm outer diameter) was used to apply traction to the mucosal flap. Lesions were deemed complex when they exceeded a total of nine points on the SMSA scoring system (size, morphology, site, and access) and recurrent when they were previously treated with endoscopic mucosal resection (EMR). Outcome measures included procedural success, total procedure time, complications, and recurrence rate at 3-month follow-up.

**Results:**

Nine patients (mean age 62.3 ± 14.5 years) were included; five had rectal and four had tumors in the sigmoid colon. The median SMSA score was 14 (SMSA Level IV—complex polyp), while three patients were pre-treated with EMR. DEA-ESD was technically feasible in all cases. En bloc resection and R0 resection rates were 100%, respectively, with a mean procedure time of 128.4 ± 54.1 min. No immediate or delayed complications occurred.

**Conclusions:**

DEA-ESD is a feasible and safe method for treating complex or recurrent tumors in the rectum and distal colon.

**Electronic supplementary material:**

The online version of this article (10.1007/s10151-020-02308-4) contains supplementary material, which is available to authorized users.

## Introduction

Endoscopic submucosal dissection (ESD) is a sophisticated resection technique for the treatment of precancerous and early cancerous lesions in the gastrointestinal tract (GIT) [1]. During ESD, endoscopic dissection of the submucosal layer is a key step towards successful en bloc and R0 resection [2]. Nevertheless, in many cases, it proves to be difficult to access the submucosal layer, especially in large, flat, or recurrent lesions following endoscopic mucosal resection (EMR) [3]. In such cases, the submucosal space is either narrow or fibrotic with difficult visualization of the cutting plane and increased risk of complications, i.e., bleeding or perforation. Widening of the submucosal space facilitating visualization and dissection of the submucosal layer during ESD can be achieved by traction, offering the potential to improve the speed and ease of submucosal dissection. Various advanced technology endoscopes, innovative devices i.e., DiLumen Endolumenal Interventional Platform (Lumendi Ltd., High Wycombe, UK) or ORISE TRS (Boston Scientific Corp, Marlborough, MA, USA), as well as techniques, i.e., clip-with-line or gravity, have been implemented in an effort to optimize traction during ESD and ultimately enhance the procedure`s performance [4]. Double-endoscope assisted ESD (DEA-ESD) achieves traction using two separate endoscopes inserted into the lumen parallel to each other. A grasping forceps advanced through the secondary endoscope is used to tug on the mucosal flap, thereby maintaining adequate view of the submucosal layer, while ESD is performed through the primary endoscope. Although this method has been proven to be efficacious in the treatment of upper gastrointestinal lesions [5], data regarding its efficacy on colorectal lesions ESD are scarce [6]. Moreover, it remains to be elucidated whether this method could be advantageous in the resection of difficult colorectal polyps. We conducted a pilot study aiming to evaluate the feasibility and safety of the DEA-ESD for the resection of colorectal tumors.

## Materials and methods

### Study population

This study was conducted from February to July 2019 in the Department of Gastroenterology, University Clinic of Augsburg, Germany. Consecutive patients aged older than 18 years were included when en bloc resection with other resection techniques, particularly EMR (lesions size > 20 mm, recurrence after EMR), was not possible or when malignancy was macroscopically suspected (depressed morphology, pit pattern V). Data were collected prospectively and analyzed retrospectively. All patients provided written informed consent. The study was conducted in accordance with the ethical principles of the Declaration of Helsinki, in compliance with good clinical practice and according to local regulations.

### Study measures and definitions

The primary outcome of the study was the procedural success of DEA-ESD (R0 resection rate, recurrence rate) [7]. Other outcome measures included procedure time (time for total ESD procedure), en bloc resection (defined as resection of the targeted area in one piece [8]), R0 resection (defined as histopathologically proven tumor-free lateral and vertical margins without lymphatic or vascular involvement and submucosal invasion depth < 1000 μm and no poorly differentiated histology [9]), resection speed (estimated in minutes/cm^2^, as previously reported [2]), and safety (complication rate as previously defined [10]). A lesion was deemed complex when it exceeded a total of nine points on the SMSA scoring system (size, morphology, site, and access) [11] and recurrent if it has been treated previously by EMR. Lesion size was calculated as the surface of an ellipse using the long and the short diameter of the resection specimen and is presented in cm^2^ [2].

### Diagnostic work-up

All lesions were initially assessed with white light as well as narrow band imaging and classified according to the Paris, laterally spreading tumors (LST) and Japan NBI Expert Team (JNET) classifications, respectively. A SMSA score was estimated for each lesion [11].

### Double-endoscope ESD procedure (DEA—ESD): Video 1

**Fig. 1:**
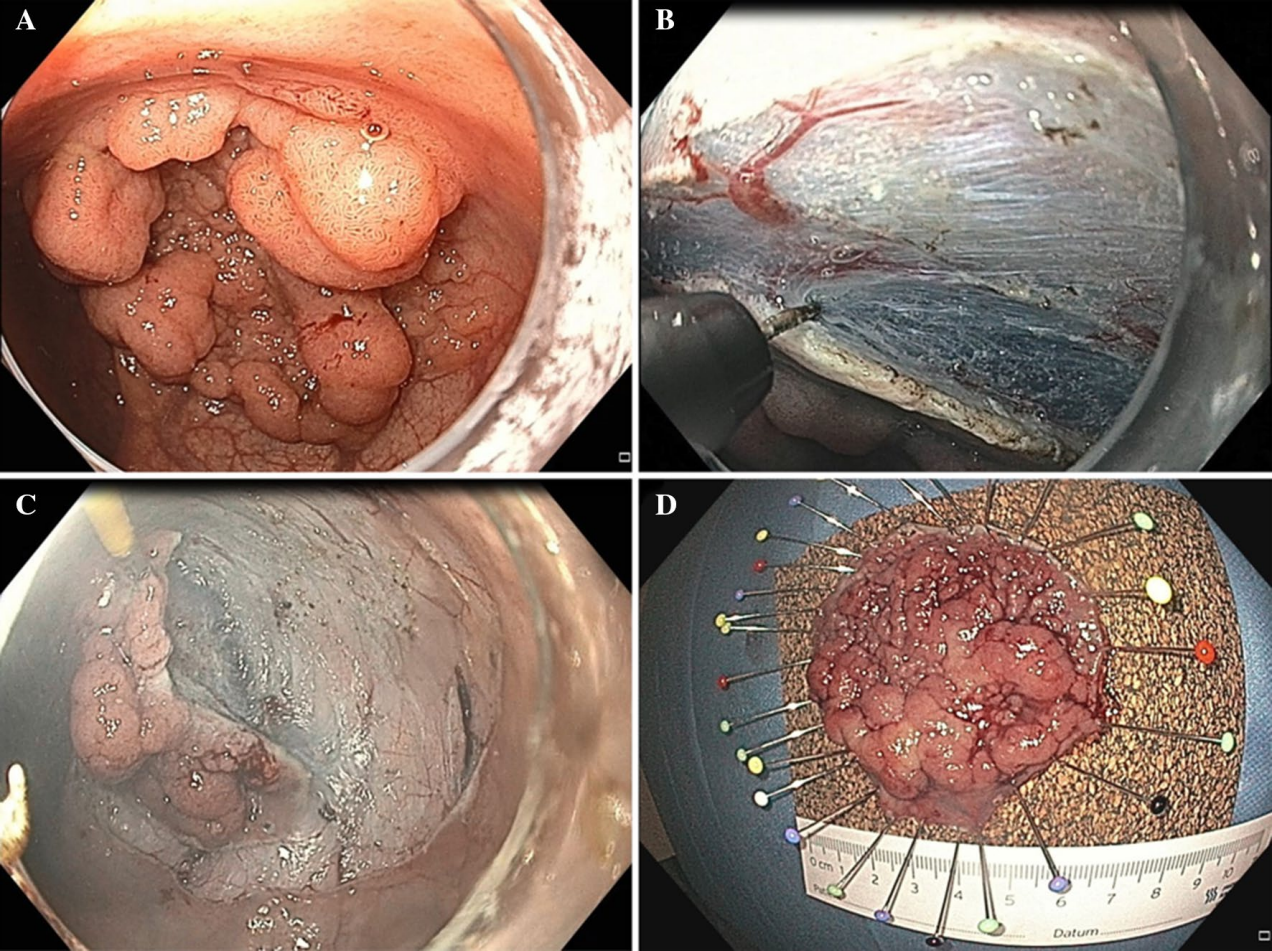
**a**, **b** Endoscopic submucosal dissection of a laterally spreading tumor in the rectum. **c** Traction applied to the lesion with use of a grasping forceps, introduced through the working channel of a small-caliber endoscope. **d** Resection specimen—histopathology assessment showed tubular adenoma with high-grade intraepithelial neoplasia resected R0

For all procedures, a video gastroscope (GIF-HQ190, Olympus Medical Systems, Tokyo, Japan) with a transparent cap (D-201–11804, Olympus) was used. Sedation included midazolam and propofol administered by a second physician, under constant cardiorespiratory monitoring. A mixed solution of saline, epinephrine (1:100.000), glycerol (10%), and indigo-carmine was used for the submucosal injection, while in cases with severe fibrosis, hyaluronic acid (Sigmavisc™, Hyaltech Ltd., Livingston, UK) was available. For all procedures, a hook knife (KD-620LR; Olympus), an electrosurgical unit (VIO 300D electrosurgical generator (ERBE Elektromedizin, Tübingen, Germany), and insufflation with carbon dioxide were used. ESD was performed in a standardized manner initially with injection, mucosal incision, and submucosal dissection at lesion’s distal margin in forward view. Thereafter, circumferential mucosal incision was completed and partial exfoliation using the primary endoscope was performed. Next, a smaller caliber endoscope (GIF-XP190N, Olympus Medical Systems, Tokyo, Japan, 5.4 mm outer diameter) using a second light source mounted on a complementary endoscopic tower and operated by a second physician was inserted into the lumen parallel to the primary endoscope. The second physician was positioned to the right side of the primary physician. The second endoscopic tower was positioned beside the primary tower, so that both physicians had a clear view of both monitor screens. It was usually helpful to insert the primary endoscope first after which the second endoscope was advanced and not vice-versa. This enabled the better positioning of both endoscopes. Friction was sometimes experienced between the scopes, especially for more proximally located lesions. Generous amounts of lubricants were always necessary and, sometimes, silicon spray was helpful in reducing friction between the scopes. A grasping forceps (Raptor® grasping device—mini, US endoscopy, Mentor OH, USA) was advanced through the working channel of the smaller caliber endoscope allowing the lesion to be grasped along its margin (Fig. 1). Each endoscope was maneuvered and controlled by the respective physician. The grasping forceps was controlled by the second physician and in some cases by a second assisting endoscopy nurse. Lesions could be grasped at the endoscopist`s discretion, either proximally in the reverse-viewing mode or distally in the forward-viewing mode. The thinner endoscope could move independently and the grasping forceps could be also inserted or retracted on demand. Traction direction was easy to control due to scope`s flexibility, while optimal visualization of the submucosal plane achieved by tissue traction resulted in safer dissection. The second endoscope was used intermittently during the procedure, and traction could be applied even after having completed the incision, and was withdrawn after sufficient entrance into the submucosal layer was achieved. For lesions with fibrotic and non-lifting mucosa, the thinner endoscope was inserted on demand, especially when areas of non-lifting were encountered. However, during dissection, the second endoscope had to be kept as motionless as possible so as not to convey movements of the second endoscope onto the movements of the primary endoscope. All ESD procedures were performed by two ESD experts (HM and AE).

### Histopathologic evaluation and follow-up

After each ESD, the specimen was fixed on cork with needles and fixed in 10% formalin. Specimen size was measured and sent for histopathological assessment. Lesions were classified as adenoma with either low-grade or high-grade intraepithelial neoplasia (LGIEN, HGIEN). R0 or R1 was described for the vertical margin (VM) and horizontal margin (HM). Follow-up with repeat endoscopy was scheduled at 3 months.

### Statistical analysis

Quantitative data were expressed as mean (standard deviation [SD]) or median with range and qualitative data, as frequencies and percentages, respectively. Statistical Package for the Social Sciences (SPSS) v.24 (IBM, Chicago, IL, USA) was used for the analysis.

## Results

A total of nine DEA-ESD procedures were performed for colorectal lesions. All procedures were technically feasible and none was discontinued during implementation of the method. Patient, tumor characteristics, and DEA-ESD outcomes are summarized in Table 1. In five patients, the tumor was located in the rectum, while in other cases, the lesions were in the sigmoid colon. Paris type 0–IIa + Is (granular type LST nodular mixed type) was the most frequent lesion type (55.6%). All lesions scored a minimum of 12 points (median 14, range 12–16) in the SMSA scoring system, and, therefore, were level IV (complex polyp), while pre-ESD endoscopic treatment (EMR) had been performed in three patients. Non-lifting sign was not evident in any lesion. DEA-ESD was technically feasible in all cases (*n* = 9, 100%). Mean tumor size was 64.02 cm^2^ (SD 31.2) with a mean procedure time of 128.4 min (SD 54.1) and mean resection speed of 2.26 min/cm^2^ (SD 1.06). En bloc and complete resection rates were 100%, respectively. No immediate or delayed complications related to the procedure occurred, while on follow-up endoscopy at 3 months, residual/recurrent tumor was not detected in any of the patients (*n* = 0, 0.0%).

**Table 1:** Patient and lesion characteristics and outcomes of treatment

Patient characteristics	
Age, mean (SD), years	62.3 (14.5)
Sex, male/female, *n* (%)	6 (66.7)/3 (33.3)
ASA grade I/II, *n* (%)	3 (33.3)/6 (66.7)
Lesion characteristics	
Location: rectum/ sigmoid colon, *n* (%)	5 (55.6)/4 (44.4)
Treatment-naïve lesion, *n* (%)	6 (66.7)
Pretreated lesion (previous EMR), *n* (%)	3 (33.3)
Paris classification: 0–Is/0–IIa/0–IIa + Is/0–IIa + IIc, *n* (%)	0/3 (33.3)/5 (55.6)/1 (11.1)
JNET classification: type 2A/Type 2B, *n* (%)	7 (77.8)/2 (22.2)
Surface pattern: granular/non granular/granular mixed	0/3 (33.3)/6 (66.7)
SMSA scoring system: median (range)	14 (12—16)
Lesion size: mean (SD), cm^2^	64.02 (31.2)
Histology: LGIEN/HGIEN, *n* (%)	5 (55.6)/4 (44.4)
Outcomes of treatment	
Successful rate of DEA-ESD, *n* (%)	9 (100.0)
Procedure time: mean (SD), minutes	128.4 (54.1)
Resection speed: mean (SD), minutes/cm^2^	2.26 (1.06)
Resection: En bloc/piecemeal, *n* (%)	9 (100.0)/0 (0.0)
R0 resection: R0/R1–Rx, n (%)	9 (100.0)/0 (0.0)
Complications (CTCAE grade)^a^, *n* (%)	0 (0.0)
Recurrence at 3 months after index ESD, *n* (%)	0 (0.0)

SD standard deviation, ASA American Association of Anesthesiologists, EMR endoscopic mucosal resection, JNET Japan NBI Expert Team, LGIEN low-grade intraepithelial neoplasia, HGIEN high-grade intraepithelial neoplasia, SMSA size, morphology, site, and access score, ESD endoscopic submucosal dissection

^a^Including delayed bleeding, perforation, stenosis, and pain CTCAE, Common Terminology Criteria of Adverse Events (version 4.0)

## Discussion

DEA-ESD has been proposed as a feasible method that could improve traction and ultimately improve ESD performance. To the best of our knowledge, this is the first study to demonstrate feasibility of the DEA-ESD for resection of recurrent and pre-defined complex lesions in the rectum and sigmoid colon in a large-volume European center.

Rectal ESD procedures may not be particularly demanding due to easy accessibility, straight scope, and the relatively thick rectal wall. However, the level of difficulty rises significantly for larger lesions spreading across folds with poor accessibility such as those located in the proximal rectum or distal sigmoid. In the absence of a validated scoring system, we used the SMSA system for stratifying the difficulty of resection. Although its ability to effectively predict colorectal ESD clinical outcomes (excessive duration of the procedure, percentage of piecemeal resections, aborted procedures, and complications) has been recently challenged, whether this also extends to other outcomes, i.e., R0 resection remains to be seen [12]. Furthermore, recurrent lesions with fibrotic submucosal tissue will show little or no lifting during submucosal injection making the dissection of the submucosal layer extremely demanding. In this regard, our findings support the notion that DEA-ESD can be a useful adjunct tool when treating complex (SMSA score > 9) lesions, as it was successfully applied in all cases. Traction provided was easily maneuverable due to the thinner scope`s increased flexibility that allowed accurate identification of the submucosal plane increasing the efficiency of dissection while, at the same time, reducing the risk of complications.

One might dispute the method`s utility given the fact that its applicability is limited to the distal colon and rectum while at the same time less resource-consuming (i.e., no need for a second endoscopist) and cost-effective methods to apply traction, i.e., clip-with-line or gravity are available. Still, for such lesions, gravity or tissue traction may not be sufficient. Moreover, expertise level among endoscopists who perform ESD may significantly vary. On this point, DEA-ESD could be potentially helpful for endoscopists with a slow learning curve, which, indeed, seems to be the case in Western countries [10]. An additional advantage of the technique as compared to other traction methods would be the possibility to apply it at any time during the ESD. Moreover, in recent years, several novel devices that provide and facilitate tissue retraction in special cases have emerged. Among them, a double balloon platform and sheath (DiLumen Endolumenal Interventional Platform; Lumendi Ltd., High Wycombe, UK) that facilitates exposure of difficult-to-access, due to poor scope maneuverability and loop presence, lesions [13], and another system (ORISE TRS; Boston Scientific Corp, Marlborough, MA, USA) that expedites colorectal ESD in cases of significant fibrosis from previous tattooing [14]. Nonetheless, these pioneer systems have not yet been extensively studied, concerns about their maneuverability have been raised, while the additional financial burden from their use cannot be underestimated.

A number of studies have investigated the applicability of DEA-ESD for GIT tumors (Table 2 summarizes data from all available studies evaluating exclusively the use of a second endoscope to deliver traction during ESD performance in various gastrointestinal tract sites [5, 6, 15–18]). However, the majority of them refer to upper GIT lesions. To date, only a single study compared ESD outcomes regarding colorectal laterally spreading tumors (LST) in 21 cases of DEA-ESD and 16 cases of standard ESD [6]. En bloc resection was similar in the two methods [21 (100%) vs. 16 (100%), p = n.s]. Although procedure time was shorter with DEA-ESD, the difference did not reach significance (96 ± 53 min vs. 116 ± 74 min, *p* = n.s). It is noteworthy that examinations were performed by ESD experts in a Japanese center; this limits generalizability of the results in every-day clinical practice. Higuchi et al. [15] in a retrospective study used a switchable light source between the two endoscopes for treating early gastric cancer. This modification improved the cutting rate into specimen (7% vs. 35%, *p* = 0.01), while no serious adverse events was noted. However, it should be underlined that all comparisons were made with historical control data, Ahn et al. [5] found that the main outcomes of DEA-ESD and standard technique used for gastric lesions did not differ. Despite its good design, the low number of patients (*n* = 51) and lack of experience by endoscopists performing the ESD are points that attracted criticism. Ogata et al. [16] enrolled more participants (*n* = 122), highlighting the safety and efficacy of a double-endoscopic intraluminal operation for precancerous gastric lesions. Finally, results from the most recent iterations show a similar duration and complication rate for DEA-ESD and standard technique when treating upper GIT lesions [17, 18]. Data from those studies show that the method is “operator friendly”, improving accessibility to the sub-mucosa, while reducing the risk of complications. However, interference between the two endoscopes and need for two light sources, endoscopes and physicians, seems to undermine its role. Moreover, data evaluating the method’s efficacy based on the anatomical site of the lesion or the endoscopist’s level of expertise are lacking. In the current study, we went a step further and evaluated DEA-ESD on tumors with a high SMSA score or recurrent lesions, showing excellent en bloc and curative resection rates with no recurrence. Of note, we did not find any limitation regarding the maneuverability of the two endoscopes in such large lesions; perhaps, the small diameter of the second scope played a significant part in this preventing friction between the scopes. Most importantly, no complications occurred, even though the expected risk of perforation and bleeding is high. Although our results suggest that DEA-ESD is feasible and safe, the small number of patients enrolled is the study’s main limitation. Moreover, all cases were performed with the DEA- ESD method, and thus, no comparison with the standard ESD technique in terms of procedure parameters, i.e., resection speed, is possible.

**Table 2:** Comparison of current study results with the literature

Author references	Uraoka et al. [6]	Ahn et al. [5]	Higuchi et al. [15]	Ogata et al. [16]	Colak et al. [17]	Sohda et al. [18]	Current study
Country	Japan	Korea	Japan	Japan	Turkey	Japan	Germany
Study design	Prospective, single-center	RCT, single center	Retrospective, single center	Prospective, single-center	Retrospective, single-center	Retrospective, single-center	Prospective, single-center
Time period	04/2006–10/2008	06/2010–08/2011	10/2008–05/2012	1999–2015	01/2014–04/2018	01/2010–06/2016	02–07/ 2019
Pts enrolled, (total/intervention/no—intervention, *n*)	37/21/16	51/25/26	57/30/27	122/122/0	22/22/0	111/51/60	9/9/0
Endoscopist expertise level	1 expert	2 nonexperts	Experts	NA	NA	Experts	2 experts
ESD location, *n*	Rectum: 14, Sigmoid: 7	Stomach, 51	Stomach, 30	Stomach, 122	Stomach, 22	Esophagus, 111	Rectum 5, Sigmoid 4
Tumor size (mean ± SD; mm)	43.6 ± 16	20.5 ± 7.9	20 median (range 2–42)	1.8 median (range 0.2–4.2)	NA	32.3 ± 11.2	52.1 ± 12.2
SMSA Score (median, range)	NA	NA	NA	NA	NA	NA	14 (12—16)
Pretreated (EMR), *n* (%)	NA	NA	NA	NA	NA	NA	3 (33.3)
En bloc resection, *n* (%)	21 (100)	26 (100)	30 (100)	119 (97.5)	NA	46 (90.0)	9 (100)
Procedure time (mean ± SD; minutes)	96 ± 53	29.2 ± 12.6	80 (range 35–201 min)	70.9 (range 20–207 min)	54 (range 45–75 min)	114 ± 54.5	128.4 ± 54.1
Complications, *n* (%)	0 (0.0)	2 (7.7)	9 (30)	7 (6.0)	NA	1 (1.96)	0 (0.0)
Recurrence, *n* (%)	NA	NA	NA	2 (1.6)	NA	NA	0 (0.0)

*RCT* randomized control trial, *Pts* patients, *ESD* endoscopic submucosal dissection, *SMSA* site, morphology, size, access scoring system, *EMR* endoscopic mucosal resection, *NA* not available

## Conclusions

DEA-ESD is a feasible and safe method for treating complex or recurrent tumors in the rectum and distal colon.

DEA-ESD can be considered by European endoscopists as an option to enhance the accessibility of the sub-mucosa as well as increase the efficiency of submucosal dissection of complex colorectal polyps. Further studies are warranted to establish the value of this method compared to other traction methods available.

## Electronic supplementary material

Below is the link to the electronic supplementary material.

Video 1. Double-endoscope assisted ESD (DEA-ESD) procedure (MP4 14220 kb)

## References

[1] Probst A, Ebigbo A, Markl B, Schaller T, Anthuber M, Fleischmann C, Messmann H (2017). Endoscopic submucosal dissection for early rectal neoplasia: experience from a European center. Endoscopy.

[2] Ebigbo A, Probst A, Rommele C, Messmann H (2018). Step-up training for colorectal and gastric ESD and the challenge of ESD training in the proximal colon: results from a German Center. Endosc Int Open.

[3] Kim JH, Nam HS, Choi CW, Kang DH, Kim HW, Park SB, Kim SJ, Hwang SH, Lee SH (2017). Risk factors associated with difficult gastric endoscopic submucosal dissection: predicting difficult ESD. Surg Endosc.

[4] Tziatzios G, Ebigbo A, Golder SK, Probst A, Messmann H (2020). Methods that assist traction during endoscopic submucosal dissection of superficial gastrointestinal cancers: a systematic literature review. Clin Endosc.

[5] Ahn JY, Choi KD, Lee JH, Choi JY, Kim MY, Choi KS, Kim DH, Song HJ, Lee GH, Jung HY, Kim JH, Baek S (2013). Is transnasal endoscope-assisted endoscopic submucosal dissection for gastric neoplasm useful in training beginners? A prospective randomized trial. Surg Endosc.

[6] Uraoka T, Ishikawa S, Kato J, Higashi R, Suzuki H, Kaji E, Kuriyama M, Saito S, Akita M, Hori K, Harada K, Ishiyama S, Shiode J, Kawahara Y, Yamamoto K (2010). Advantages of using thin endoscope-assisted endoscopic submucosal dissection technique for large colorectal tumors. Dig Endosc.

[7] Probst A, Ebigbo A, Markl B, Ting S, Schaller T, Anthuber M, Fleischmann C, Messmann H (2018). Endoscopic submucosal dissection for rectal neoplasia extending to the dentate line: European experience. Endosc Int Open.

[8] Probst A, Schneider A, Schaller T, Anthuber M, Ebigbo A, Messmann H (2017). Endoscopic submucosal dissection for early gastric cancer: are expanded resection criteria safe for Western patients?. Endoscopy.

[9] Tanaka S, Kashida H, Saito Y, Yahagi N, Yamano H, Saito S, Hisabe T, Yao T, Watanabe M, Yoshida M, Kudo SE, Tsuruta O, Sugihara K, Watanabe T, Saitoh Y, Igarashi M, Toyonaga T, Ajioka Y, Ichinose M, Matsui T, Sugita A, Sugano K, Fujimoto K, Tajiri H (2015). JGES guidelines for colorectal endoscopic submucosal dissection/endoscopic mucosal resection. Dig Endosc.

[10] Probst A, Golger D, Anthuber M, Markl B, Messmann H (2012). Endoscopic submucosal dissection in large sessile lesions of the rectosigmoid: learning curve in a European center. Endoscopy.

[11] Sidhu M, Tate DJ, Desomer L, Brown G, Hourigan LF, Lee EYT, Moss A, Raftopoulos S, Singh R, Williams SJ, Zanati S, Burgess N, Bourke MJ (2018). The size, morphology, site, and access score predicts critical outcomes of endoscopic mucosal resection in the colon. Endoscopy.

[12] JM-G (2019) Is the SMSA Score accurate enough to preoperatively predict suboptimal clinical outcomes in colorectal endoscopic submucosal dissection (CR-ESD)? A multicenter Spanish prospective study. UEG Week Barcelona, Spain 2019

[13] Jacques J, Albouys J, Guyot A, Geyl S, Legros R, Chaput U, Pioche M (2019). Endoscopic submucosal dissection of a laterally spreading tumor in the right colon with a gastroscope after shortening the colon using a new double-balloon platform. Endoscopy.

[14] Jawaid S, Yang D, Draganov PV (2019). Tissue retractor system-assisted endoscopic submucosal dissection of a large rectal tumor with significant fibrosis from direct tattooing. VideoGIE.

[15] Higuchi K, Tanabe S, Azuma M, Sasaki T, Katada C, Ishido K, Naruke A, Mikami T, Koizumi W (2013). Double-endoscope endoscopic submucosal dissection for the treatment of early gastric cancer accompanied by an ulcer scar (with video). Gastrointest Endosc.

[16] Ogata K, Yanai M, Kuriyama K, Suzuki M, Yanoma T, Kimura A, Kogure N, Toyomasu Y, Ohno T, Mochiki E, Kuwano H (2017). Double endoscopic intraluminal operation (DEILO) for early gastric cancer: outcome of novel procedure for endoscopic submucosal dissection. Anticancer Res.

[17] Çolak Ş, Gürbulak B, Çakar E, Bektaş H (2019). Resection of Mucosal and Submucosal Gastrointestinal Lesions and a Double EndoscopeExperience. JSLS.

[18] Sohda M, Kuriyama K, Yoshida T, Kumakura Y, Honjo H, Sakai M, Miyazaki T, Kuwano H (2020). Comparable Data Between Double Endoscopic Intraluminal Operation and Conventional Endoscopic Submucosal Dissection for Esophageal Cancer. J Gastrointest Surg.

